# Retraction: ‘HOXD-AS1 confers cisplatin resistance in gastric cancer through epigenetically silencing PDCD4 via recruiting EZH2’ (2019), by Ye *et al.*

**DOI:** 10.1098/rsob.230322

**Published:** 2023-09-08

**Authors:** Open Biology editorial team

**Keywords:** PDCD4, lncRNA, HOXD-AS1, gastric cancer

*Open Biol.* **9**, 190068. (Published online 25 September 2019). (doi:10.1098/rsob.190068)

The Editor-in-Chief in agreement with the Publisher has retracted this article. Following an investigation, we have found that the published manuscript has substantial overlap with published manuscript [1] in the text and figures (notably the graphs presented in figure 1 ‘HOXD-AS1 was increased in gastric cancer tissues and cells’ and figure 5 ‘Tumour images ‘HOXD-AS1 silencing enhanced DDP sensitivity in vivo’). This paper has been identified among of a set of papers that present significant text and data duplication and therefore the data reported in this article are unreliable. The authors have been unable to provide the original data requested and are in agreement with this retraction. Corresponding author Liang Ming (mengqihou820@gmail.com)

—Prof. Jon Pines FRS Editor-in-Chief, *Open Biology*
